# Water-Dispersible Supramolecular Nanoparticles Formed by Dicarboxyl-bis-pillar[5]arene/CTAB Host–Guest Interaction as an Efficient Delivery System of Quercetin

**DOI:** 10.3390/ijms27010516

**Published:** 2026-01-04

**Authors:** Marco Milone, Martina Mazzaferro, Antonella Calderaro, Giuseppe T. Patanè, Davide Barreca, Salvatore Patanè, Norberto Micali, Valentina Villari, Anna Notti, Melchiorre F. Parisi, Ilenia Pisagatti, Giuseppe Gattuso

**Affiliations:** 1 Dipartimento di Scienze Chimiche, Biologiche, Farmaceutiche ed Ambientali, Università degli Studi di Messina, Viale F. Stagno d’Alcontres 31, 98166 Messina, Italy; marco.milone@unime.it (M.M.); martina.mazzaferro@studenti.unime.it (M.M.); anto.calderaro@gmail.com (A.C.); giuseppe.patane@studenti.unime.it (G.T.P.); dbarreca@unime.it (D.B.); anotti@unime.it (A.N.); mparisi@unime.it (M.F.P.); 2 Dipartimento di Chimica, Biologia e Biotecnologie, Università degli Studi di Perugia, Via Elce di Sotto, 8, 06123 Perugia, Italy; 3 Dipartimento di Scienze Matematiche e Informatiche, Scienze Fisiche e Scienze della Terra, Università degli Studi di Messina, Viale F. Stagno d’Alcontres 31, 98166 Messina, Italy; patanes@unime.it; 4 CNR-IPCF Istituto per i Processi Chimico-Fisici, Viale F. Stagno d’Alcontres 37, 98158 Messina, Italy; norbertoliborio.micali@cnr.it (N.M.); villari@ipcf.cnr.it (V.V.)

**Keywords:** pillar[5]arenes, supramolecular nanoparticles, quercetin, antioxidant activity, DOSY NMR, drug delivery

## Abstract

Supramolecular nanoparticles offer an efficient strategy to enhance the solubility, stability, and bioavailability of poorly water-soluble therapeutic molecules. In this study, water-dispersible SNPs were successfully prepared from dicarboxyl-bis-pillar[5]arene (**H**) and cetyltrimethylammonium bromide (CTAB) using a microemulsion method. Dynamic light scattering revealed that the resulting CTAB/**H** nanoparticles possessed a size distribution centered around 40 nm, a positive surface charge (+15 mV), and exhibited high colloidal stability over three months. ^1^H NMR, 2D TOCSY, 2D NOESY, diffusion ordered NMR spectroscopy, and UV-Vis investigations confirmed the inclusion of the CTAB alkyl chain within the pillar[5]arene cavity, supporting the formation of stable supramolecular assemblies capable of efficiently encapsulating the poorly water-soluble flavonol quercetin (**Q**). The CTAB/**H** system displayed low cytotoxicity (up to 50 µg/mL) and pronounced antioxidant activity, as evidenced by DPPH, ABTS, and FRAP assays. Quercetin-loaded nanoparticles (CTAB/**H**/**Q**) enhanced cellular uptake and exhibited a marked cytoprotective effect against H_2_O_2_-induced oxidative stress in NIH-3T3 fibroblasts.

## 1. Introduction

Nanoparticle engineering [1] has emerged as an efficient strategy for targeted drug delivery, enabling the precise targeting of specific cells while minimizing side effects, protecting sensitive therapeutics from premature degradation and enhancing the solubility of challenging-to-deliver drugs [2]. The microemulsion method has proven to be a versatile tool extensively investigated for producing water-dispersible supramolecular nanoparticles for the delivery of poorly water-soluble drugs. They offer a variety of advantages including ease of preparation, thermodynamic stability, and the ability to significantly enhance drug bioavailability [3]. Additionally, microemulsion-produced supramolecular nanoparticles (SNPs) exhibit a high solubilization capacity for both lipophilic and hydrophilic drugs, effectively protecting incorporated drugs from oxidative and enzymatic degradation [4]. The small particle size of SNPs facilitates drug absorption and distribution due to their high surface area and enhanced permeability [5]. This well-established approach has been successfully applied to the solubilization, transport, and delivery of many different compounds [6]. Among these, commercially available microemulsion formulations of cyclosporin A [7], saquinavir [8], and ritonavir [8] exemplify the successful application of this technique.

Quercetin [9] is a natural phenolic compound belonging to the flavonols family [10,11], primarily occurring in plant tissues as a glycoside. It possesses notable antioxidant properties [12] and has shown significant potential as a protective agent against cancer [13], along with a range of therapeutic benefits, including antibacterial, anti-diabetic, anti-inflammatory, and antiviral effects [14]. However, the poor solubility (0.00215–0.00263 g/L [15]), low bioavailability (24 ± 9% [16]), and limited stability of quercetin toward oxidation [17] have limited its applications in biomedicine [18]. The encapsulation of quercetin in water-dispersible SNPs offers a practical solution to these challenges [19,20].

Pillar[*n*]arenes represent an emerging family of macrocyclic hosts that have gained prominence due to their unique symmetrical pillar-like structure [21,22]. Their low cytotoxicity has drawn attention to their potential health-related applications, including biomaterials and pharmaceutics [23,24]. However, pillar[*n*]arenes suffer from intrinsically low aqueous solubility (due to their hydrophobic aromatic framework) which limits guest encapsulation efficiency and restricts biological applications [25]. Their compatibility with aqueous systems requires post-synthetic chemical functionalization: ionic and hydrophilic functional groups can be introduced onto the rims of the macrocycle scaffold to improve water solubility [26]. Such modifications increase electrostatic repulsion between macrocycles and broaden the range of operability of pillararenes host–guest chemistry through combined hydrophobic and ionic interactions [27]. A wide range of architectures can be built from pillararenes by incorporating different amphiphiles: pillararenes act both as hosts forming supramolecular amphiphiles via host–guest complexation and as components of amphiphilic supramolecular polymer conjugates. These host–guest-based supramolecular amphiphiles can further self-assemble into diverse nanoaggregates, such as vesicles, micelles, and nanoparticles, opening the way to stimuli-responsive control over encapsulation and release [28,29,30,31,32,33,34,35,36]. Notably, reports on pillararene-based recognition and/or delivery of flavonoids are scarce, likely due to steric mismatch with the macrocycle narrow cavity and unfavorable stereoelectronic interactions between these electron-rich aromatic species [18,37].

A variety of SNPs have been developed using water-soluble pillararenes with stimuli-responsive functional groups and appropriate guest species, leading to the self-assembly of amphiphilic complexes that form stimuli-responsive nanostructures [38,39,40]. However, there are still relatively few SNPs based on lipophilic pillar[*n*]arenes that do not involve amphiphilic self-assembly [41,42,43]. Recently, it was demonstrated that SNPs based on a bis-pillar[5]arene compound obtained via the microemulsion method may act as carrier matrix for a representative anticancer drug, exhibiting dual stimuli-responsiveness towards both spermine and glutathione [44]. Another study [45] reported nanoparticles composed of a bis-pillar[5]arene complex with a boron-dipyrromethene (BODIPY)-based photosensitizer capable of generating superoxide radical (O_2_^−•^) for photodynamic therapy.

**Figure 1:**
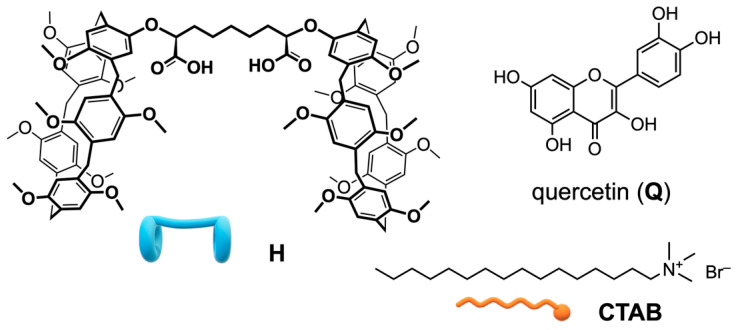
Structures of the compounds used in this study.

As part of our ongoing research on proton-ionizable [46] pillararene-based supramolecular polymers [47,48], we recently reported the synthesis of a novel bis-pillar[5]arene dicarboxylic acid (**H**, Figure 1) that can self-assemble, in the presence of complementary partners such as 1,12-dodecanediyl-bis-1,1′-1*H*-imidazole and bis-*N*,*N*’-(6-(1H-imidazole)decyl)-perylene bisimide, to yield stimuli-responsive copolymeric aggregates. These aggregates can act as efficient off-on luminescent sensing agents for the cancer marker spermine, both in solution and in solid films. Building on our earlier investigations into drug-delivery systems based on water-soluble macrocycles [49,50,51], we decided to explore the recognition properties of this receptor in an aqueous environment. Our objective was to characterize the physical properties and biological activities of new water-dispersible SNPs prepared using the microemulsion method, focusing on the host–guest interaction between cetyltrimethylammonium bromide (CTAB) and **H** to gain structural insights into their self-assembly. It was envisaged that the carboxyl (or carboxylate) moiety may facilitate the formation of CTAB/**H** SNPs, promoting CTAB inclusion in the pillararene cavity by providing additional electrostatic interactions with the charged cetyltrimethylammonium head. Further investigations have been carried out to demonstrate the potential of using dicarboxyl-bis-pillar[5]arene-CTAB colloidal nanoparticles as a promising drug delivery system for quercetin (**Q**).

## 2. Results and Discussion

### 2.1. Preparation and Characterization of Supramolecular Nanoparticles (SNPs)

SNPs were prepared using a microemulsion method from dicarboxyl-bis-pillar[5]arene **H** [41], following the oil-in-water (O/W) solvent evaporation technique. A 200 µL solution of **H** (20 mM) in CHCl_3_ was added to 10 mL of an aqueous solution of CTAB (0.9 mM, slightly below the critical micelle concentration (cmc), 0.94 mM [52]) followed by probe sonication for 25 min without temperature control (to allow the evaporation of CHCl_3_). The successful fabrication of SNPs was confirmed by ^1^H and diffusion ordered spectroscopy (DOSY) NMR, dynamic light scattering (DLS) and zeta-potential analysis.

**Figure 2:**
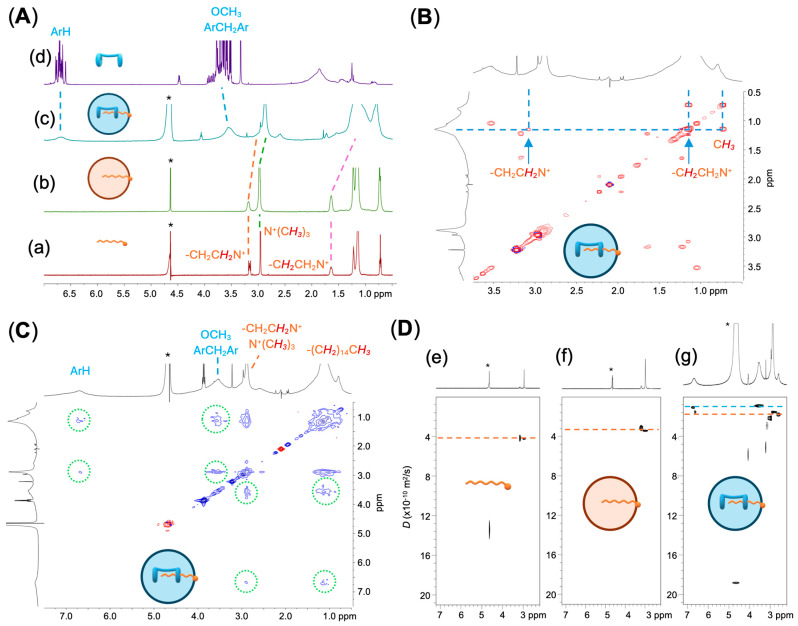
(Panel (**A**)): ^1^H NMR spectra (500 MHz, D_2_O, 25 °C) of (**a**) [CTAB] = 0.9 mM, (**b**) SNPs of [CTAB] = 0.9 mM and (**c**) CTAB/**H** SNPs ([CTAB] = 0.9 mM, [**H**] = 0.4 mM); (**d**) ^1^H NMR spectrum (500 MHz, CDCl_3_, 25 °C) of [**H**] = 5 mM. (Panel (**B**)): section of the 2D TOCSY NMR spectrum (500 MHz, D_2_O, 25 °C) of CTAB/**H** SNPs ([CTAB] = 0.9 mM, [**H**] = 0.4 mM). (Panel (**C**)): 2D NOESY NMR spectrum (500 MHz, D_2_O, 25 °C) of CTAB/**H** SNPs ([CTAB] = 0.9 mM, [**H**] = 0.4 mM). (Panel (**D**)): sections of the DOSY plots and the ^1^H NMR spectra (500 MHz, 25 °C, D_2_O) of (**e**): [CTAB] = 0.9 mM; (**f**) SNPs of [CTAB] = 0.9 mM, and (**g**) CTAB/**H** SNPs ([CTAB] = 0.9 mM, [**H**] = 0.4 mM). * Asterisks indicate the suppressed HDO solvent peak.

The ^1^H NMR spectrum of CTAB ([CTAB] = 0.9 mM, D_2_O) displayed sharp resonances characteristic of a monomeric species. In contrast, the ^1^H NMR spectra of the SNPs prepared from CTAB and the CTAB/**H** system revealed a remarkably different profile compared with CTAB on its own. As shown in Figure 2, panel A, the resonances of the emulsified CTAB broadened, indicating an aggregation process, thereby confirming its surfactant nature. Remarkably, when the same emulsification process was applied to the CTAB/**H** system, new broad resonances appeared at δ 6.68 and 3.52 ppm (assigned to the aromatic hydrogen atoms and methoxy/bridging methylene hydrogens, respectively [48]), consistent with the presence of bis-pillar[5]arene **H** in the colloidal solution. Furthermore, the small but significant complexation-induced shifts observed for specific resonances of CTAB, assigned by a 2D TOCSY experiment (Figure 2, panel B), suggest a self-assembly process, wherein the aliphatic chain of CTAB likely resides within the cavity of the pillar[5]arene, experiencing favorable CH–π interactions, while the hydrophilic alkylammonium head of CTAB pointing towards the bulk solvent. This hypothesis was confirmed by a 2D NOESY experiments (Figure 2, panel C), which provided through-space NOE correlations between the aromatic hydrogen atoms, the methoxy and the bridging methylenes of **H** on one hand, and the methyl and methylene hydrogens of the included cetyltrimethylammonium guest on the other.

Additional insights into the self-assembly of the CTAB/**H** system were gathered by means of comprehensive DOSY studies carried out before and after emulsification (Figure 2, panel D) [53]. The self-diffusion coefficient (*D*) was derived from the decay of the resonances assigned to the trimethylammonium head of CTAB. Prior to the microemulsion process, CTAB on its own showed a *D* = 4.41 ± 0.02 × 10^−10^ m^2^/s. After emulsification, this value decreased to 3.41 ± 0.02 × 10^−10^ and further reduced to 1.56 ± 0.01 × 10^−10^ m^2^/s in the CTAB/**H** system. The self-diffusion coefficient measured for the resonance of the aromatic hydrogen atoms of **H** was *D* = 1.29 ± 0.02 × 10^−10^ m^2^/s.

The decrease in the self-diffusion coefficients for CTAB suggests the transition from freely diffusing monomers to aggregates, even below the cmc of the surfactant. The colloidal properties of ionic single-tail surfactants can be influenced by the addition of organic additives [54,55]. The resulting mixed components display aggregating features (e.g., lower cmc) different from those of the individual molecules, as a result of a combination of electrostatic (polar heads) and hydrophobic/van der Waals (lipophilic tails) interactions between the two molecules [56]. Similar reductions in *D* are systematically reported when guest molecules or macrocyclic hosts bind to surfactant assemblies, where the measured diffusion coefficient represents an average over fast exchange between free and bound states [57]. Therefore, the decrease in *D* for CTAB, approaching the value observed for **H**, indicates the incorporation into a common supramolecular entity, leading to the formation of stable SNPs [58,59].

**Figure 3:**
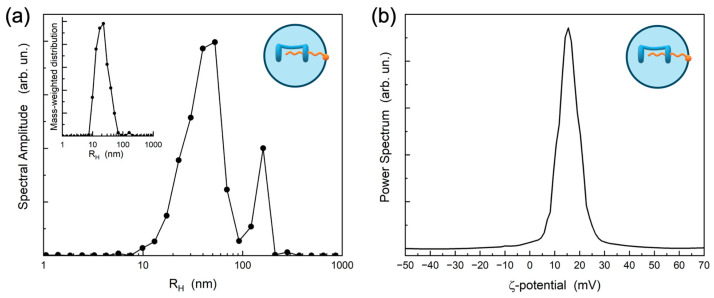
(**a**) Intensity-weighted size distribution at the scattering angle of 90° (the size distributions at different scattering angles were analogous) and (**b**) ζ-potential of CTAB/**H** SNPs. The inset of plot (**a**) displays the mass-weighted size distribution obtained by using the Mie scattering model [60].

For the CTAB/**H** system, DLS results (Figure 3 and Figure S1, see Supplementary Material) revealed a predominant size distribution, with an average hydrodynamic radius (R_H_) of approximately 40 nm, while zeta-potential analysis yielded a positive value of +15 mV, indicating a positive surface charge of the SNPs. The secondary peak in the intensity-weighted size distribution at larger hydrodynamic radii could originate from some nanoparticle clusters. However, the mass-weighted size distribution, reported in the inset of Figure 3a, reveals that the amount of such clusters is negligible with respect to the main nanoparticle family, suggesting that the nanoparticle size meets the homogeneity generally required for drug delivery applications. Furthermore, these values remained stable over three months at 4 °C, revealing an excellent stability of the colloidal nanoparticles. The small size, uniform size distribution, and long-term stability suggest that these SNPs are suitable for drug delivery applications.

**Figure 4:**
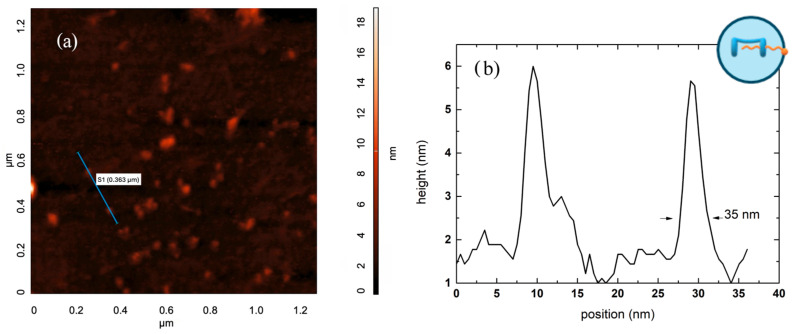
(**a**) AFM morphology of the sample surface, showing well-defined, flattened nanoparticles with an approximately circular shape and lateral dimensions between 20 and 40 nm. (**b**) Line profile acquired along the path indicated in (**a**).

Morphological analysis was carried out using Atomic Force Microscopy (AFM) [49]. The resulting surface morphology (Figure 4a) shows well-defined, flattened nanoparticles with an approximately circular shape, whose lateral size ranges from about 20 to 50 nm. The nanostructures exhibit a maximum height of about 5–6 nm, indicating that they are colloidal nanoparticles whose typically spherical shape was altered (i.e., collapsed) during the dehydration process required for AFM sample preparation. Figure 4b displays the line profile acquired along the path drawn in Figure 4a, highlighting the presence of particles with an average lateral size of about 25 nm. As expected for this type of sample, a distribution in particle sizes is observed, consistent with its intrinsic nature. It is important to note that the line-profile analysis only probes a limited number of nanoparticles, which may not provide a statistically representative sample of the entire surface. Notably, the lateral size distribution exhibits a tail extending toward larger diameters, with values approaching ~70–90 nm (Figures S2 and S3). This observation supports the consistency between AFM and DLS measurements, as these larger lateral dimensions are compatible, considering particle flattening, with the hydrodynamic size obtained from DLS (hydrodynamic radius ~40 nm).

**Figure 5:**
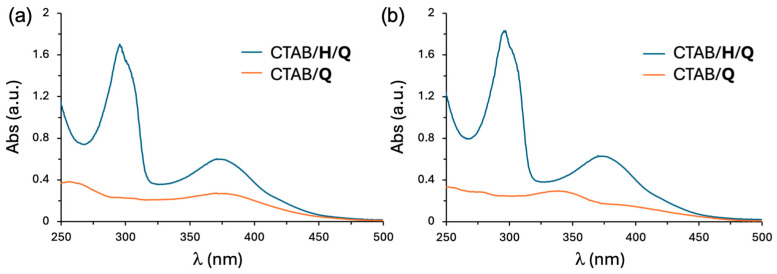
UV/Vis spectra of: (**a**) CTAB/**H**/**Q** ([CTAB] = 6.00 × 10^−5^ M; [**H**] = 2.67 × 10^−5^ M; [**Q**] = 2.11 × 10^−5^ M) and CTAB/**Q** ([CTAB] = 6.00 × 10^−5^ M; [**Q**] = 9.43 × 10^−6^ M) formulation freshly prepared and (**b**) the same CTAB/**H**/**Q** and CTAB/**Q** formulations after 7 days.

To assess the intracellular drug delivery potential of the SNPs, quercetin **Q** was selected as a representative poorly water-soluble cargo molecule. CTAB and CTAB/**H** SNPs were employed for solid–liquid extraction experiments using a fixed aliquot of **Q** (1 mg). After 12 h stirring at room temperature, the resulting suspensions were centrifuged to remove undissolved quercetin, and the supernatants were subjected to UV-Vis analyses (Figure 5). As shown in Figure 5a, quercetin was successfully loaded into both CTAB and CTAB/**H** SNPs, as indicated by the absorption band at 370 nm, characteristic of the Band I of the cinnamoyl system of quercetin, with the CTAB/**H** system showing a stronger absorption.

In addition, the colloidal solution of CTAB/**H** SNPs displayed a distinct band at 290 nm, providing evidence of the presence of the pillararene skeleton. UV/vis data indicated that the quercetin concentrations after the extraction were [**Q**] = 2.11 × 10^−5^ M (corresponding to a loading capacity (LC%) of 9.5% [61] and an encapsulation efficiency (EE%) of 95.6% [62]) and 9.43 × 10^−6^ M in the CTAB/**H**/**Q** and CTAB/**Q** systems, respectively. As illustrated in Figure 5b, the CTAB/**H**/**Q** formulation retained its stability even after 7 days, whereas significant changes were observed in the CTAB/**Q** SNPs, potentially attributed to aggregation/oxidation phenomena that may reduce **Q** bioavailability [63].

### 2.2. Biological Activity

**Figure 6:**
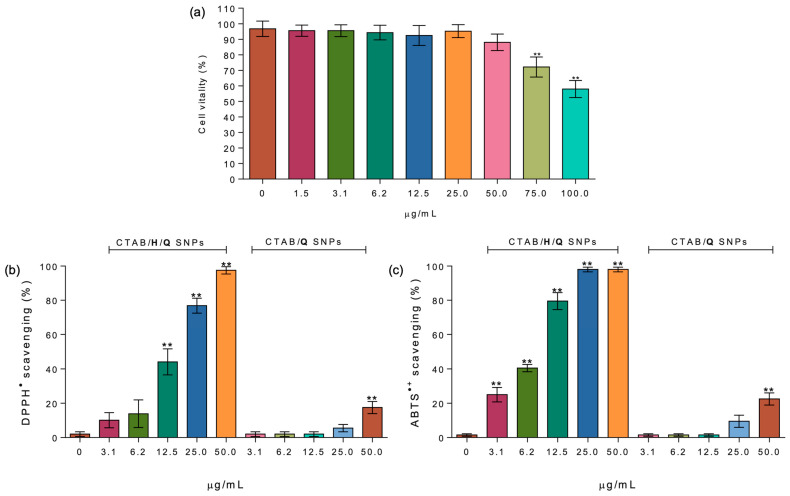
(**a**) Cytotoxicity assays. Viability of NIH-3T3 cells after 24 h incubation with different concentrations of CTAB/**H** SNPs. Cell viability was determined using the MTT assay. Data are expressed as mean ± SD, with asterisks (**) indicating significant statistical difference at *p* < 0.05. (**b**) Antioxidant activity of CTAB/**H**/**Q** SNPs against DPPH. (**c**) Antioxidant activity of CTAB/**H**/**Q** SNPs against ABTS radical. Statistical analysis was performed using one-way analysis of variance (ANOVA) and the significance of differences from respective controls was assessed using Dunnett’s test.

A mitochondrial cytotoxic test was employed to assess the cytotoxicity of the CTAB/**H** SNPs system, aiming to preliminarily establish its potential as a carrier for poorly soluble drugs in living systems. Cell vitality was determined using the thiazolyl blue tetrazolium bromide (MTT) assay [64] on NIH-3T3 cells. As depicted in Figure 6a, CTAB/**H** SNPs exhibited no significant difference in cell vitality compared to the control at concentrations up to 50.0 µg/mL. However, at higher concentrations (75.0 and 100.0 µg/mL), a substantial decrease in cell vitality was observed, with reductions of approximately 27% and 58%, respectively.

The antioxidant ability of CTAB/**H**/**Q** SNPs, in the 0.0–50.0 µg/mL concentration range, was evaluated using two widely recognized radical scavenging assays (DPPH and ABTS), as well as a ferric-reducing antioxidant power (FRAP) assay. These assays encompass the two primary mechanisms by which antioxidants neutralize free radicals [65]: electron transfer (ET) and hydrogen atom transfer (HAT) for DPPH and ABTS, respectively. In the DPPH assay, the CTAB/**H**/**Q** SNPs were able to completely scavenge the radicals at the maximum tested concentration (50.0 µg/mL), while scavenging ability decreased to approximately 44 and 77% at 12.5 and 25.0 mg/mL, respectively. The lowest concentrations tested (6.25 and 3.12 µg/mL) showed antioxidant activities ranging between 10 and 14%. The EC_50_ of the CTAB/**H**/**Q** system determined from the DPPH assay (Figure 6b) is 17.84 µg/mL. Considering a quercetin loading capacity of 9.5%, this corresponds to an effective **Q** concentration of approximately 1.69 µg/mL. This value is in excellent agreement with the EC_50_ of free quercetin (5.4 µM in ethanol, equivalent to 1.63 µg/mL [66]), indicating that the delivery system does not significantly alter quercetin activity and allows **Q** to exhibit its full DPPH scavenging ability in aqueous medium.

Data obtained for the scavenging of the ABTS radical cation by CTAB/**H**/**Q** SNPs showed complete radical quenching in the 25.0–50.0 µg/mL concentration range, with scavenging activities of approximately 79, 40, and 25% observed at 12.5, 6.25, and 3.12 µg/mL, respectively. This difference in the antioxidant capacity between the two assays may be attributed to both the use of different solvents (methanol vs. water) and the nature of the radical (neutral vs. cationic) being scavenged. As reported in Figure 6b,c, the antioxidant activity exhibited by quercetin-loaded CTAB/**Q** SNPs is either absent or significantly lower than that obtained with CTAB/**H**/**Q** SNPs at all the tested concentrations in both antioxidant assays. In the FRAP assay, CTAB/**H**/**Q** SNPs at a concentration of 25.0 µg/mL displayed a ferric-reducing antioxidant power corresponding to 5.70 ± 0.09 µM Trolox equivalents, whereas CTAB/**Q** SNPs at the same concentration showed a substantially lower value of 0.47± 0.04 µM Trolox equivalents, reflecting the greater content of available redox-active quercetin in the CTAB/**H**/**Q** SNPs.

**Figure 7:**
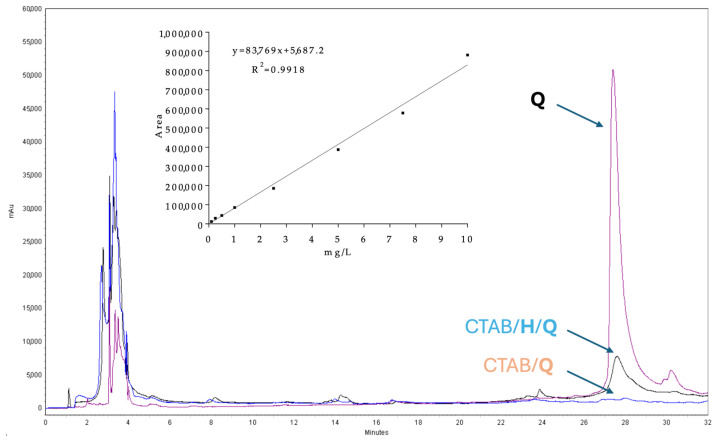
Representative chromatogram for the identification and quantification of cellular uptake. The chromatograms were recorded at λ = 258 nm. **Q**: standard reference obtained with pure quercetin; CTAB/**H**/**Q**: recovery of quercetin after cellular uptake with quercetin-loaded CTAB/**H**/**Q** SNPs; CTAB/**Q**: recovery of quercetin after cellular uptake with quercetin-loaded CTAB/**Q** SNPs. Inset: calibration curve for quercetin (**Q**) quantification.

Having assessed the non-cytotoxicity of CTAB/**H** SNPs and their remarkable antioxidant activity, quercetin-loaded CTAB/**H**/**Q** SNPs were assayed for their ability to act as a cellular drug delivery system and their cytoprotective effect against H_2_O_2_-induced oxidative damage. NIH-3T3 were incubated with 50 μg/mL of CTAB/**H**/**Q** SNPs (final concentration) and the cellular uptake of quercetin was analyzed by RP-HPLC-DAD. As depicted in Figure 7, quercetin showed a well-defined peak with a retention time (R_t_) of 27.45 min. Quantitative analysis revealed a quercetin uptake of 7.10 ± 0.27 μg/mL per 5 × 10^5^ cells, significantly higher than that obtained with quercetin-loaded CTAB/**Q** SNPs (0.33 ± 0.07 μg/mL per 5 × 10^5^ cells) used as a control.

**Figure 8:**
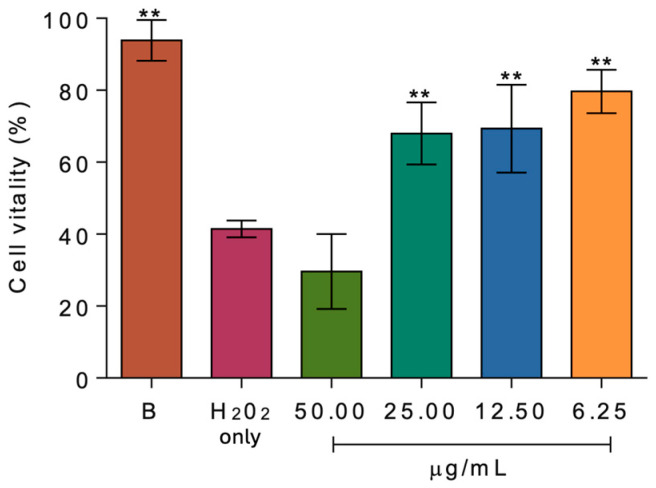
Cytoprotective assays. Viability of NIH-3T3 cells after 5 h incubation with H_2_O_2_ in the absence or presence of different concentrations of CTAB/**H**/**Q** SNPs. Cell viability was determined using the MTT assay. Data are expressed as mean ± SD, with asterisks (**) indicating significant statistical difference at *p* < 0.05. Statistical analysis was performed using one-way analysis of variance (ANOVA), and the significance of differences from respective controls was assessed by using Dunnett’s test.

The protective activity of CTAB/**H**/**Q** against oxidative stress injury was determined in NIH-3T3 cells. As shown in Figure 8, the H_2_O_2_ induced a significant decrease in cell viability (~58.5%). Preincubation with CTAB/**H**/**Q**, at the concentration of 6.2, 12.5 and 25.0 µg/mL, markedly reduced the loss of cells vitality (~21, 30, and 32%, respectively), highlighting the cytoprotective potential of the **Q**-loaded SNPs. Surprisingly, at higher concentration (50.0 µg/mL) CTAB/**H**/**Q** acted as a pro-oxidant, further reducing cells vitality (up to ~70%) compared to treatment with H_2_O_2_ alone.

## 3. Materials and Methods

### 3.1. General Experimental

Bis-pillar[5]arene **H** was prepared according to a literature procedure [48]. Quercetin (>95% HPLC), cetyltrimethylammonium bromide (CTAB), deuterium oxide, chloroform-*d*_3_, HPLC-grade chloroform, Dulbecco’s Modified Eagle Medium (DMEM), inactivated bovine serum, penicillin G, streptomycin, trypsin, phosphate buffer (PBS), 3-(4,5-dimethylthiazol-2-yl)-2,5 diphenyltetrazolium bromide (MTT), 2,2-diphenylpyrylhydrazyl (DPPH), FeCl_3_, 2,4,6-tris(2-pyridyl)-s-triazine (TPTZ), hydrochloric acid, 2,2′-azino-bis-(3-ethylbenzothiazoline-6 sulphonic) acid (ABTS^●+^), 2,2-dimethyl-2-silapentane-5-sulfonate sodium salt (DSS), ammonium peroxydisulfate, dimethyl sulfoxide (DMSO, puriss. p.a., dried ≤0.02% water), HPLC-grade water, HPLC-grade acetonitrile, HPLC-grade methanol were supplied by Merck Life Science s.r.l., Milan, Italy, and used as received. Murine fibroblast NIH-3T3 cells (cat. no. CRL-1658) were purchased from ATCC, Rockville, MD, USA. NMR spectra were recorded at 25 °C in D_2_O or CDCl_3_ on a Varian 500 MHz instrument (Palo Alto, CA, USA) equipped with a broadband pulse-field gradient probe. Chemical shifts are reported in ppm and are referenced to the CHCl_3_ residual solvent peak (δ = 7.26 ppm) or to 2,2-dimethyl-2-silapentane-5-sulfonate sodium salt (DSS) as an external standard. ^1^H NMR spectra in D_2_O were recorded using a solvent suppression pulse sequence (PRESAT).

### 3.2. Preparation of Supramolecular Nanoparticles (SNPs)

SNPs were prepared using the microemulsion solvent evaporation method [41]. A 200 µL solution of **H** (20 mM) in CHCl_3_ was injected into 10 mL of an aqueous solution of CTAB (0.9 mM) followed by probe sonication for 25 min (Qsonica LLC, Newtown, CT, USA).

### 3.3. Stability Study

The prepared SNPs were stored in refrigerator at 4 °C for 3 months to assess any potential aggregation. The stability of the formulation was evaluated by monitoring changes in size distribution, zeta-potential, and quercetin entrapment efficiency.

### 3.4. Preparation of Quercetin-Loaded SNPs

Quercetin-loaded SNPs were prepared by solid–liquid extraction of 1 mg of quercetin with 0.8 mL of SNP colloidal solution. The supersaturated mixture was stirred vigorously using a magnetic stirrer for 12 h and then centrifuged (Prism Mini Centrifuge, Labnet, Edison, NJ, USA) for 20 min at 2000 rcf to separate the undissolved **Q**. Quercetin loading and encapsulation were quantified by UV/vis spectroscopy, comparing the results to calibration curves obtained from a commercially available standard with known concentration (0.1–10.0 mg/L). Loading capacity (LC%) was calculated according to the following equation [61]:

(1) $$LC\%=\left(\frac{weight\;of\;quercetin\;in\;nanoparticles}{weight\;of\;nanoparticles}\right)\times 100$$

Encapsulation efficiency (EE%) was calculated as follows [62]: 3 mg of **Q** were suspended in 3 mL of D_2_O and stirred vigorously using a magnetic stirrer for 12 h and then centrifuged for 20 min at 2000 rcf. The concentration of **Q** in the supernatant was determined by UV/vis spectroscopy, comparing the results to calibration curves, and calculated according to a standard curve. EE% was calculated to be 95.6% according to the following equation:

(2) $$EE\%=\left(\frac{free\;dissolved\;quercetin}{total\;dissolved\;quercetin}\right)\times 100$$

### 3.5. Two-Dimensional TOCSY, 2D NOESY and DOSY NMR

Two-dimensional ^1^H–^1^H TOCSY spectra were acquired at 25 °C with a solvent suppression sequence, using a MLEV-17 spin-lock sequence with a mixing time of 150 ms, 8 transients for each increment (1024 in total). Spectra were collected with a spectral width of 10 ppm in both dimensions. Two-dimensional ^1^H–^1^H NOESY experiments were carried out at 25 °C with a solvent suppression sequence using a 500 ms mixing time, 16 transients for each increment (512 in total) and a relaxation time of 3 s. Spectra were collected with a spectral width of 10 ppm in both dimensions. Diffusion-ordered NMR spectroscopy (DOSY) studies were performed at 25 °C using a DgcsteSL pulse sequence, with a solvent suppression sequence, optimizing experimental parameters according to the sample under investigation. Diffusion gradients were progressively incremented over 15–30 steps, varying the gradient strength from 1.7 to 54.0 gauss cm^–1^. 64–512 transients were acquired for each increment, with a diffusion-gradient length of 2 ms and diffusion delays in the 100–200 ms range. Spectra were recorded with a 10 ppm spectral width (from –2 to 8 ppm), and a 1 s relaxation delay. Diffusion coefficients were averaged from three experiments.

### 3.6. Dynamic Light Scattering

The size distribution of the nanoparticles was determined by using a custom-built Photon Correlation Spectroscopy set-up, consisting of a vertically polarized He-Ne laser source (35 mW), i.e., orthogonal to the scattering plane, focused on the sample, two single-photon counting avalanche photodiodes (in pseudo-cross-correlation mode) and a Malvern correlator (System 4700c, Malvern Instruments Ltd., Worcestershire, UK) for collecting the intensity-intensity correlation functions in a standard VV geometry. The scattering angle was varied in the range 45–110°. The SNP solution was diluted ten times in pure water to a final concentration of [CTAB] = 0.09 mM and [**H**] = 0.04 mM. The measured pH was 6.9 ± 0.1 and the ionic strength was 1 mM. Temperature was set at 22 °C. The CONTIN algorithm was used to obtain the intensity-weighted the size distribution. Further details have been reported elsewhere [67,68].

### 3.7. Electrophoretic Light Scattering (Photon Correlation Spectroscopy)

The particle zeta-potential (ζ) measurements were carried out using a Brookhaven ZetaPALS (Holtsville, NY, USA) instrument, which employs two different methods to assess the particle electrophoretic mobility: the conventional laser Doppler electrophoresis based on the frequency shift in the scattered beam with respect to the reference one, and the principle of Phase Analysis Light Scattering (PALS), which analyses the phase change between the Doppler signal and the reference signal. The measured electrophoretic mobility (µ) was related to the zeta-potential by using the Henry equation:

(3) $$\zeta =3\mu \eta /\left[2\varepsilon f\left(\kappa R\right)\right]$$

where ε and η are the dielectric constant and viscosity of the solvent, respectively, κ is the Debye–Hückel constant, R is the particle radius, with 1 ≤ f(κR) ≤ 1.5. Both methods were used and gave the same results. More detail can be found elsewhere [69,70]. The solution of the SNPs was diluted in pure water ten times in a glass cell (to a final concentration of [CTAB] = 0.09 mM and [**H**] = 0.04 mM) in which an electrode (BI-SR367 by Brookhaven) was inserted. The measured pH was 6.9 ± 0.1 and the ionic strength was adjusted to 1 mM with the addition of NaCl. Temperature was set at 22 °C. The measurements were performed in triplicate for each method (repeated on two fresh samples) and, due to the small statistical deviation, the average value was calculated.

### 3.8. Atomic Force Microscopy (AFM)

CTAB/**H** thin films for AFM characterization were prepared at room temperature by spin-coating a 5 × 10^−6^ M aqueous dispersion onto silica substrates at approximately 1000 rcf. Following deposition, the samples were dried under vacuum for 30 min. AFM measurements were performed using an NT-MDT SMENA (Zelenograd, Moscow, Russia) instrument equipped with a silicon probe operated in semi-contact mode (NSG30, nominal tip radius ~6 nm). The AFM images were subsequently processed using a plane-removal algorithm to eliminate background and tilt.

### 3.9. Cell Culture

Murine fibroblast NIH-3T3 cells, cultured at 37 °C in humidified 5.0% CO_2_ atmosphere in DMEM supplemented with 10% (*v*/*v*) inactivated bovine serum (FBS), penicillin G and streptomycin (100 units/mL and 100 μg/mL, respectively). Before a uniform monolayer of cells was formed, cells were detached from the culture flask surface using a 0.25% trypsin solution with EDTA and subcultured two to three times weekly.

### 3.10. Cytotoxicity Assay

200 μL of NIH-3T3 cells were seeded into 96-well plates at density of 1 × 10^4^ cells/well. After a 24 h incubation and attachment period, cells were treated with varying concentrations (0.0–100.0 µg/mL, where 100 µg/mL corresponds to [CTAB] = 90.3 µM, [**H**] = 40.2 µM and [**Q**] = 2.1 µM) of the tested formulation. The plates were incubated at 37 °C in a 5% CO_2_ atmosphere for 24 h. Following incubation, the medium was carefully aspirated, and the plates washed three times with 200 μL of PBS (20 mM, pH = 7.3). Cells were then incubated for an additional 3 h with DMEM (without serum) containing 0.01% MTT. After incubation, the medium was aspirated and the MTT crystals dissolved with 100 µL of DMSO. The plates were then gently shaken to solubilize the formazan, and absorbance was measured at 570 nm [64] with a microplate reader (Accuris™ SmartReader™ 96, Benchmark Scientific MR9600-E-UK, Sayreville, NJ, USA). The cytotoxicity test was carried out in quintuplicate biological replicates derived each from triplicate technical replicates and expressed as mean ± standard deviation (SD). Cell viability (%) was calculated as follows:

(4) $$Cell\;viability\;\left(\%\right)=\left(\frac{A_{S}}{A_{C}}\right)\times 100$$

where A_S_ is the absorbance of the samples treated with the tested compound and A_C_ is the absorbance of the untreated samples.

### 3.11. Cellular Uptake

2.0 mL of NIH-3T3 cells was seeded into 6-well plates at density of 2 × 10^5^ cells/well. Following a 24 h incubation and attachment period, the cells were treated with a final concentration of 50.0 µg/mL of the tested formulation. The plates were incubated at 37 °C in a 5% CO_2_ atmosphere for 4 h. After the incubation period, the medium was carefully aspirated, and cells were washed three times with 2.0 mL of PBS (20 mM, pH = 7.3) and subjected to trypsinization. The suspended cells were collected in a 2.0 mL test tube and centrifuged at 2000 rcf for 10 min. After centrifugation, the supernatant was discarded, and the remaining cells were homogenized with 300 µL of DMSO using a Microson ultrasonic cell disruptor XL (Qsonica LLC, Newtown, CT, USA), operating at 30% maximum power in cycles of 30 s followed by 30 s of pause, repeated three times. After the final sonication cycle, samples were centrifuged at 18,000 rcf for 10 min. The supernatant was filtered through an Iso-Disc P-34 (Supelco, Bellefonte, PA, USA), 3 mm diameter PTFE membrane (3 mm diameter, 0.45 μm pore size) and analyzed by reverse phase high performance liquid chromatography. Quercetin identification was carried out using Reverse Phase–High Performance Liquid Chromatography–Diode Array Detection (RP-HPLC-DAD). The column used was a BioDiscovery C18 (250.0 mm × 4.6 mm i.d., 5.0 μm, Merck/Supelco, Darmstadt, Germany) equipped with a 20.0 mm × 4.0 mm guard column. The temperature was set at 25 °C with a flow-rate of 1.0 mL/min. Separation was achieved using a linear gradient of acetonitrile in H_2_O as the mobile phase. The gradient was as follows: 0–15 min (5–20% of CH_3_CN), 15–20 min (20–30% of CH_3_CN), 20–35 min (30–100% of CH_3_CN), 35–40 min (100% of CH_3_CN), 40–45 min (100–5% of CH_3_CN), and 45–55 min (5% of CH_3_CN). Chromatograms were recorded at 258 nm and UV/vis spectra were collected between 200 and 450 nm. Compound identification was based on retention time, UV spectra, and co-elution with authentication standards. Quantitative analysis was conducted by integrating the areas of the peaks from the chromatogram at 258 nm and comparing them with calibration curves obtained from a commercially available standard (in the 0.1–10.0 mg/L range).

### 3.12. Cytoprotective Assay Against Oxidative Damage Induced by H_2_O_2_

The assay was carried out by following the procedure described by Lin et al. [71]. 200 μL of NIH-3T3 cells was seeded into 96-well plates at a density of 1 × 10^4^ cells/well. Following a 24 h incubation and attachment, cells were pretreated with varying concentrations (6.25, 12.50, 25.00, and 50.00 μg/mL) of CTAB/**H**/**Q** SNPs for 2 h, followed by induction of cellular damage with H_2_O_2_ (800 μM) for 5 h. After incubation, the medium was carefully aspirated, and cells were washed three times with 200 µL of PBS (20 mM, pH = 7.3). Cells were then incubated for an additional 3 h with DMEM (without serum) containing 0.01% MTT (final concentration). After incubation, the medium was aspirated and the MTT crystals dissolved with 100 µL of DMSO. The plates were then gently shaken to solubilize the formazan, and absorbance was measured at 570 nm [64] with a microplate reader. The cytotoxicity test was performed in quintuplicate biological replicates derived each from triplicate technical replicates and expressed as mean ± standard deviation (SD).

### 3.13. 2,2-Diphenylpyrylhydrazyl (DPPH) Radical Assay

The antioxidant activity of CTAB/**H**/**Q** SNPs was evaluated using the DPPH radical assay following the protocol reported by Barreca et al. [72]. In detail, an 80.0 μM DPPH was prepared, and dilutions were made with methanol to achieve a final absorbance value of 1.0. The antioxidant activity of CTAB/**H**/**Q** SNPs was analyzed in the 0.0–50.0 µg/mL concentration range. After a 30 min incubation, absorbance was measured at 517 nm, using a Varian Cary 50 UV-Vis spectrophotometer (Varian Instruments, Palo Alto, CA, USA). The antioxidant activity, expressed as radical scavenging activity (%) was calculated using the following equation:

(5) $$DPPH\cdot scavenging\;\left(\%\right)=\frac{\left[\left(Ac-As\right)\right]}{Ac}\times 100$$

where A_C_ represents the absorbance of the control and A_S_ the absorbance of the sample. All tests were carried out in triplicate and expressed as mean ± standard deviation (SD).

### 3.14. Ferric-Reducing Antioxidant Power (FRAP) Assay

The FRAP assay was performed following the protocol reported by Barreca et al., with minor modifications [73]. The fresh working FRAP reagent was prepared by mixing 25 mL of acetate buffer (300 mM, pH 3.6), 2.5 mL of TPTZ solution (10 mM in 40 mM HCl) and 2.5 mL of FeCl_3_ solution (20 mM) and stored at room temperature until use. The antioxidant activity of CTAB/**H**/**Q** SNPs was analyzed in the 0.0–50.0 µg/mL concentration range, with a final volume of 800 μL. After 4 min of incubation under gentle shaking, absorbance was recorded at 595 nm using a Varian Cary 50 UV-Vis spectrophotometer. All experiments were carried out in triplicate. Antioxidant activity is expressed as μM Trolox equivalent.

### 3.15. ABTS Assay

The antioxidant activity of CTAB/**H**/**Q** SNPs against the ABTS^●+^ radical cation was assessed following a method reported by Papalia et al. [74]. A solution of 7.0 mM ABTS and 10.0 mM ammonium peroxydisulfate was mixed in phosphate buffer (pH 7.2) and allowed to react overnight. Appropriate dilutions were made to achieve absorbance value of 1.0 ta 734 nm. The activity of CTAB/**H**/**Q** SNPs was analyzed over a concentration range of 0.0–50.0 µg/mL in a final volume of 1.0 mL. After 6 min of incubation under gentle shaking, absorbance was recorded at 734 nm using a Varian Cary 50 UV-Vis spectrophotometer. Each analysis was performed in triplicate, and results were expressed as % cation radical elimination.

### 3.16. Statistical Analysis

Data are presented as means ± standard deviation (S.D.). Statistical analysis was performed using one-way analysis of variance (ANOVA) with GraphPad Prism 6.0 software (GraphPad Software, San Diego, CA, USA). The significance of differences from respective controls for each experimental test was assessed using Dunnett’s test for each paired experiment. A *p*-value < 0.05 was considered statistically significant.

## 4. Conclusions

In summary, this pilot study reports the successful preparation of stable, water-dispersible dicarboxyl-bis-pillar[5]arene/CTAB supramolecular nanoparticles that exhibit a uniform size distribution and long-term colloidal stability. These SNPs efficiently encapsulate and deliver the poorly water-soluble flavonoid quercetin, enhancing its stability and cellular uptake. Furthermore, the system demonstrates low cytotoxicity and remarkable antioxidant and cytoprotective activity against H_2_O_2_-induced oxidative stress. There are very few reports in the literature on pillararene–flavonoids interactions: this is, to the best of our knowledge, the first report on the use of a pillararene-based carrier to achieve intracellular delivery of quercetin.

## Data Availability

The raw data supporting the conclusions of this article will be made available by the authors on request.

## Supplementary Materials

The following supporting information can be downloaded at: https://www.mdpi.com/article/10.3390/ijms27010516/s1.

## Abbreviations

The following abbreviations are used in this manuscript:

ABTS	2,2′-azino-bis(3-ethylbenzothiazoline-6-sulfonic acid)
AFM	Atomic Force Microscopy
ANOVA	Analysis of variance
ATCC	American Type Culture Collection
BODIPY	4,4-difluoro-4-bora-3a,4a-diaza-*s*-indacene
CTAB	Cetyltrimethylammonium bromide
*D*	Self-diffusion coefficient
DAD	Diode Array Detection
DLS	Dynamic Light Scattering
DMEM	Dulbecco’s Modified Eagle Medium
DMSO	Dimethyl sulfoxide
DOSY	Diffusion-Order Spectroscopy
DPPH	2,2-diphenyl-1-picrylhydrazyl
ET	Electron transfer
FRAP	Ferric-Reducing Antioxidant Power
HAT	Hydrogen atom transfer
LC%	Drug loading capacity
MTT	3-(4,5-dimethylthiazol-2-yl)-2,5-diphenyltetrazolium bromide
PALS	Phase Analysis Light Scattering
PBS	Phosphate-Buffered Saline
PTFE	Polytetrafluoroethylene
Rcf	Relative Centrifugal Field
R_H_	Average hydrodynamic radius
R_t_	Retention time
RP-HPLC	Reverse Phase–High Performance Liquid Chromatography
SNPs	Supramolecular nanoparticles
TPTZ	2,4,6-Tripyridyl-s-triazine
