# Supplementary material for: Water-Dispersible Supramolecular Nanoparticles Formed by Dicarboxyl-bis-pillar[5]arene/CTAB Host–Guest Interaction as an Efficient Delivery System of Quercetin
Source: Int J Mol Sci. 2026 Jan 4;27(1):516. doi: 10.3390/ijms27010516 (PMC12786710; doi:10.3390/ijms27010516)
Supplement: Supplementary file 1 [file ijms-27-00516-s001.zip › ijms-4020863-supplementary.pdf]

International Journal of *Molecular Sciences*

MDPI

Supplementary Material

Water-dispersible supramolecular nanoparticles formed by di-carboxyl-bis-pillar[5]arene/CTAB host–guest interaction as an efficient delivery system of quercetin

Marco Milone [1,†], Martina Mazzaferro [1,2,†], Antonella Calderaro [1, †], Giuseppe T. Patanè [1], Davide Barreca [1], Salvatore Patanè [3], Norberto Micali [4], Valentina Villari [4], Anna Notti [1], Melchiorre F. Parisi [1], Ilenia Pisagatti [1,*] and Giuseppe Gattuso [1,*]

[1] Dipartimento di Scienze Chimiche, Biologiche, Farmaceutiche ed Ambientali, Università degli Studi di Messina, Viale F. Stagno d'Alcontres 31, 98166 Messina, Italy; mmilone@unime.it (M.Mi.); martina.mazzaferro@studenti.unime.it (M.Ma.); anto.calderaro@gmail.com (A.C.); giuseppe.patane@studenti.unime.it (G.T.P.); dbarreca@unime.it (D.B.); anotti@unime.it (A.N.); mparisi@unime.it (M.F.P).

[2] Dipartimento di Chimica, Biologia e Biotecnologie, Università degli Studi di Perugia, via Elce di Sotto, 8, 06123 Perugia, Italy.

[3] Dipartimento di Scienze Matematiche e Informatiche, Scienze Fisiche e Scienze della Terra, Università degli Studi di Messina, Viale F. Stagno d'Alcontres 31, 98166 Messina, Italy; patanes@unime.it (S.P.)

[4] CNR-IPCF Istituto per i Processi Chimico-Fisici, Viale F. Stagno d'Alcontres 37, 98158, Messina, Italy; norbertoliborio.micali@cnr.it (N.M.); villari@ipcf.cnr.it (V.V.).

* Correspondence: ipisagatti@unime.it (I.P.); ggattuso@unime.it (G.G.)

† These authors contributed equally to this work.

Content

Figure S1. Intensity-intensity correlation function of CTAB/H nanoparticles.
Figure S2. Morphology acquired on a different part of the sample with respect to Figure 4a.
Figure S3. Number of particles versus diameter as observed in Figure S2.

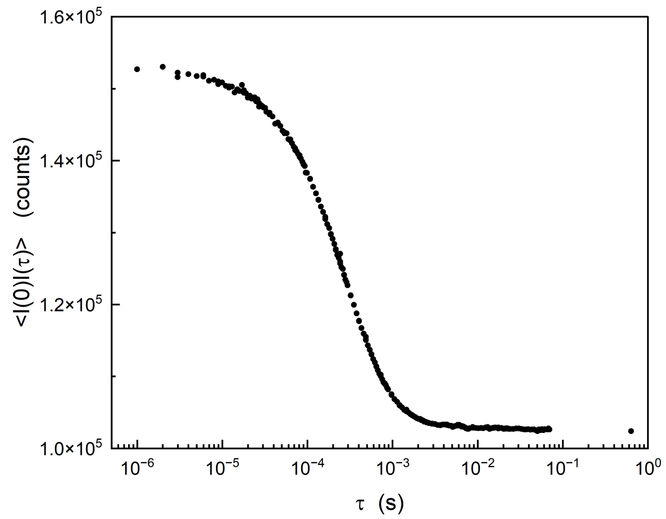

Figure S1. Intensity-intensity correlation function of CTAB/**H** nanoparticles.

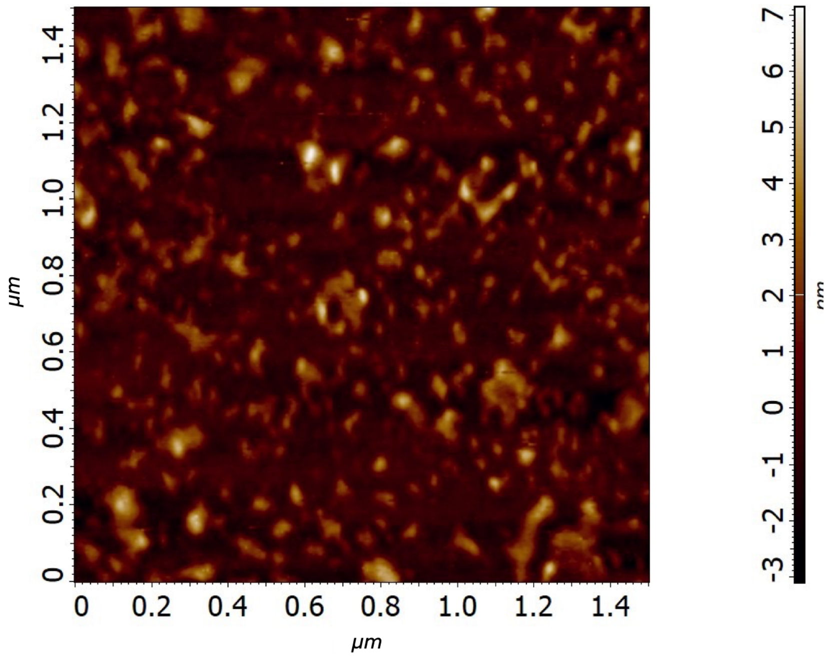

Figure S2. Morphology acquired on a different part of the sample with respect to Figure 4a.

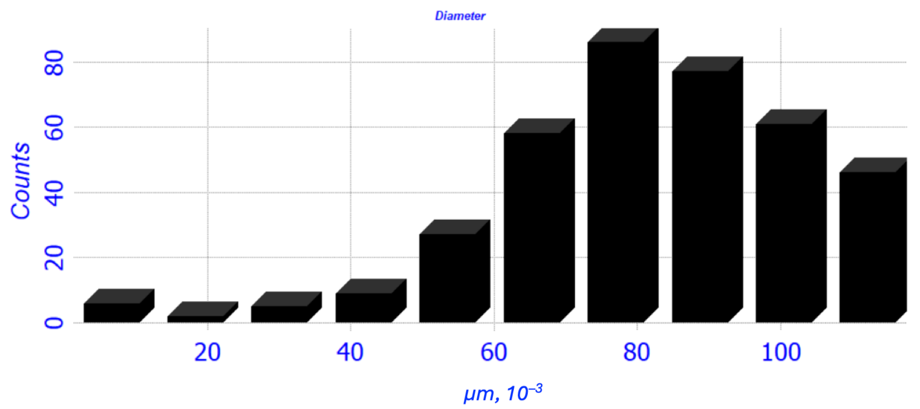

Figure S3. Number of particles versus diameter as observed in Figure S2.
